# What Is the Relationship Between Hippocampal Neurogenesis Across Different Stages of the Lifespan?

**DOI:** 10.3389/fnins.2022.891713

**Published:** 2022-05-24

**Authors:** Allison M. Bond, Guo-li Ming, Hongjun Song

**Affiliations:** ^1^Department of Neuroscience and Mahoney Institute for Neurosciences, Perelman School of Medicine, University of Pennsylvania, Philadelphia, PA, United States; ^2^Department of Cell and Developmental Biology, Perelman School of Medicine, University of Pennsylvania, Philadelphia, PA, United States; ^3^Department of Psychiatry, Perelman School of Medicine, University of Pennsylvania, Philadelphia, PA, United States; ^4^Institute for Regenerative Medicine, Perelman School of Medicine, University of Pennsylvania, Philadelphia, PA, United States; ^5^The Epigenetics Institute, Perelman School of Medicine, University of Pennsylvania, Philadelphia, PA, United States

**Keywords:** embryonic neurogenesis, adult neurogenesis, neural stem cells, lifespan, species differences

## Abstract

Hippocampal neurogenesis has typically been studied during embryonic development or in adulthood, promoting the perception of two distinct phenomena. We propose a perspective that hippocampal neurogenesis in the mammalian brain is one continuous, lifelong developmental process. We summarize the common features of hippocampal neurogenesis that are maintained across the lifespan, as well as dynamic age-dependent properties. We highlight that while the progression of hippocampal neurogenesis across the lifespan is conserved between mammalian species, the timing of this progression is species-dependent. Finally, we discuss some current challenges in the hippocampus neurogenesis field, and future research directions to address them, such as time course analysis across the lifespan, mechanisms regulating neurogenesis progression, and interspecies comparisons. We hope that this new perspective of hippocampal neurogenesis will prompt fresh insight into previous research and inspire new directions to advance the field to identify biologically significant ways to harness the endogenous capacity for neurogenesis in the hippocampus.

## Introduction

**FIGURE 1:**
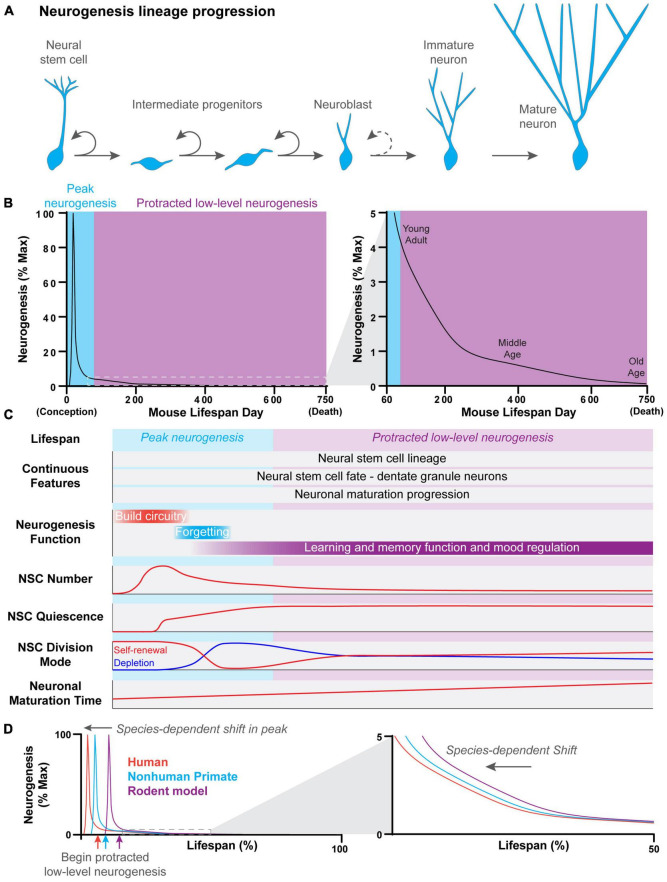
Features of hippocampal neurogenesis across the lifespan. **(A)** Newborn neurons in the dentate gyrus of the hippocampus are generated through a common process across the lifespan. Neural stem cells divide to give rise to rapidly dividing intermediate progenitor cells. Intermediate progenitors give rise to neuroblasts, which have a limited capacity to divide before terminally differentiating and transitioning into immature neurons. Immature neurons have a distinct physiology which allows them to play a unique role in the hippocampal circuitry. This distinct physiology is lost as immature neurons undergo further synaptic development and reach the mature neuron stage. **(B)** The developmental progression of continuous dentate gyrus neurogenesis across the lifespan of a mouse. A robust and acute peak in neurogenesis occurs very early in the lifespan, around birth. Peak neurogenesis is followed by a dramatic exponential decline in neurogenesis which leads into the beginning of a protracted period of low-level neurogenesis that spans most of the lifespan. Neurogenesis levels continue to exponentially decline with age, albeit at a slower rate, during the protracted period of low-level neurogenesis. **(C)** Summary of continuous features and age-dependent properties of hippocampal neurogenesis across the lifespan in mice. While features such as neural stem cell lineage, cell fate, and neuronal maturation progression remain c across the lifespan, other features, such as neural stem cell properties, the function of neurogenesis, and neuronal maturation time, change in an age-dependent manner. **(D)** The developmental progression of dentate gyrus neurogenesis across the lifespan is conserved across species. However, the timing of peak neurogenesis and the start of the protracted period of low-level neurogenesis are shifted in a species-dependent manner.

Neurogenesis is the process by which new neurons are generated from neural stem cells (NSCs) (Figure 1A; Gage, 2019). Neurogenesis peaks during early development, when new neurons are generated to build the neural circuitry that supports brain function. Neurogenesis ceases in most regions of the mammalian brain following development. However, low levels of neurogenesis continue throughout life to generate new neurons for the olfactory bulb and the dentate gyrus (DG) region of the hippocampus (Figure 1B). In the adult brain, populations of quiescent NSCs in the ventricular-subventricular zone (V-SVZ) of the lateral ventricles and the subgranular zone (SGZ) of the DG re-enter cell cycle or reactivate to generate new olfactory bulb interneurons and dentate granule neurons, respectively (Ming and Song, 2011; Bond et al., 2015). This process has historically been referred to as “adult neurogenesis,” because it was first discovered in adult rodent models. As a result, “adult neurogenesis” has been compared to “embryonic neurogenesis,” and each process has been studied as a distinct phenomenon. It was not until the last decade that researchers have begun to explore the developmental origins of adult neurogenesis and the link between developmental and adult neurogenesis (Li et al., 2013; Fuentealba et al., 2015; Furutachi et al., 2015; Berg et al., 2019).

In this perspective, we focus on the relationship between hippocampal neurogenesis across multiple stages of the lifespan in mammals, including embryonic and postnatal development, as well as adulthood and aging. We propose that hippocampal neurogenesis is one life-long developmental process with common features that remain consistent across the lifespan, as well as age-dependent properties, with focused discussion of evidence from mouse studies. We provide an in-depth discussion of the implications of this new perspective that neurogenesis is one life-long process as it relates to the purpose of protracted neurogenesis and its impact on brain function, species-related differences in neurogenesis, and the controversial topic of the existence of neurogenesis in the adult human hippocampus. Finally, we discuss current challenges and perspectives that we hope will guide future directions in hippocampal neurogenesis research.

## Hippocampal Neurogenesis Is One Continuous Developmental Process With Common Features Across the Lifespan

There has been an explosion of studies interrogating regulators of NSCs and neurogenesis in the DG region of the adult hippocampus in the last 25 years. However, the relationship between embryonic and adult hippocampal neurogenesis was not well-understood and has only recently become a topic of research (Berg et al., 2018; Bond et al., 2021). Neurogenesis in the DG is a protracted process, that in rodents begins during early embryonic brain development, peaks neonatally and continues at low levels throughout adulthood (Altman and Das, 1965; Angevine, 1965; Bayer, 1980a,b; Bond et al., 2020). Traditionally, the field has separated DG neurogenesis into embryonic and adult neurogenesis, despite its apparently continuous nature. We propose that DG neurogenesis is best studied as one continuous developmental process because new neurons originate from a common neural stem cell lineage and mature through a common lineage progression process across the lifespan.

Until recently it was unclear whether the precursors responsible for DG neurogenesis during development and adulthood shared a common lineage or were distinct precursor populations. A genetic fate-mapping study using *Gli-CreER* mice showed that precursors responding to Sonic Hedgehog (Shh) at embryonic day 17.5 (E17.5) give rise to both DG neurons and quiescent NSCs in the adult DG, suggesting that developmental and adult neurogenesis may arise from the same progenitor population (Li et al., 2013). Clonal lineage tracing using the *Hopx-CreER* mouse line provided direct evidence that a single NSC, which generates DG neurons embryonically, also generates multiple quiescent adult NSCs that continue generating DG neurons throughout adulthood (Berg et al., 2019). This result suggested that a common neural precursor population continuously contributes to neurogenesis in the DG from early embryonic development through adulthood (Figure 1C). Intriguingly, this NSC lineage remains fate restricted, generating progeny exclusively for the DG, but not other brain regions (Berg et al., 2019). In addition, NSCs from this common lineage consistently express HOPX and exhibit core transcriptomic and open chromatin signatures across the lifespan (Berg et al., 2019; Borrett et al., 2022). In contrast, precursors to NSCs in the adult mouse V-SVZ appear to generate neurons for the cortex, striatum, or septum embryonically before changing fate in their adult state to generate neurons for the olfactory bulb (Fuentealba et al., 2015). Differences in the embryonic origin of the SGZ and V-SVZ adult NSC populations and their properties suggest that there may be more than one way to maintain a long-term population of NSCs in the mammalian brain.

During mouse development, DG neurogenesis begins when NSCs divide to give rise to intermediate progenitor cells (IPCs), which expand to generate neuroblasts (Figure 1A). Proliferating neuroblasts then give rise to immature neurons, which gradually integrate into the circuitry to ultimately become mature DG neurons. Adult precursors with astroglial-like properties were shown to give rise to neurons in the adult DG, similar to radial glia cells during development, and were aptly named radial glia-like NSCs (RGLs) (Seri et al., 2001; Bonaguidi et al., 2011). Later, stage-specific markers were shown to be expressed in a similar sequence to the neurogenic lineage progression found during development (Brandt et al., 2003; Brown et al., 2003; Filippov et al., 2003; Kronenberg et al., 2003; Kempermann et al., 2004; Hevner et al., 2006; Shin et al., 2015). Adult-born neurons also followed a similar synaptic integration process to developmentally-born neurons, first receiving dendritic GABAergic inputs, then glutamatergic afferents, and finally perisomatic GABAergic inputs (Esposito et al., 2005; Ge et al., 2006). Ultimately, mature dentate granule neurons have similar afferent connectivity and exhibit similar firing patterns regardless of when they are born during the lifespan (Laplagne et al., 2006). Most recently, single-cell RNA-sequencing analysis confirmed that DG neurogenesis progresses through molecularly identical cellular states from IPCs to neuroblasts to immature DG neurons regardless of their birthdate within the lifespan (Hochgerner et al., 2018). Thus, newborn DG neurons progresses through a remarkably similar maturation process across the lifespan.

## Age-Dependent Properties of Hippocampal Neurogenesis

Some cellular properties of NSCs and newborn neurons change across the lifespan, directly impacting the rate of neurogenesis (Figure 1C). High levels of neurogenesis are required for rapid DG morphogenesis during development, while relatively low levels of neurogenesis are sufficient to modify the hippocampal circuitry postnatally. Varying levels of neurogenesis across age differentially contribute to learning and memory function. For example, moderate levels of neurogenesis during the juvenile postnatal period promote forgetting or “infantile amnesia” (Akers et al., 2014; Travaglia et al., 2016; Guskjolen et al., 2017), while the low levels of neurogenesis that occur during adulthood promote pattern separation and reduce memory interference (Figure 1C; Christian et al., 2014; Miller and Sahay, 2019; Toda et al., 2019). Thus, changes in cellular properties that affect the rate of neurogenesis likely underlie the changing functional role of neurogenesis across the lifespan.

The proliferative and self-renewal properties of DG NSCs also change in an age-dependent manner. NSCs in the developing DG are highly proliferative, in contrast to NSCs in the adult DG, which are largely quiescent. DG NSCs undergo a transition from a dividing to quiescent state during the first postnatal week of development in mice, peaking at postnatal day 3 (P3) (Figure 1C; Berg et al., 2019; Noguchi et al., 2019; Bond et al., 2020). As a result, neurogenesis rapidly declines, ending peak neurogenesis (Figure 1B). The number of NSCs decreases with age, and the NSC population becomes increasingly quiescent, contributing to the age-related decline in neurogenesis observed in adulthood (Encinas et al., 2011; Berg et al., 2019; Harris et al., 2021; Ibrayeva et al., 2021). Interestingly, the rate of decline in neurogenesis slows with advancing age, likely due to changes in NSC division mode. NSCs divide to deplete in the juvenile and young adult brain (∼0.5–6 months), while NSC division becomes more self-renewing with age (≥6 months) such that NSCs are less likely to deplete after division (Figure 1C; Harris et al., 2021; Ibrayeva et al., 2021). This change in NSC division mode compensates for the reduced NSC pool and is an example of how age-dependent cellular changes may compensate for declining neurogenesis. Newborn DG neurons also develop through a remarkably similar process no matter when they are born during the lifespan. However, the tempo of neuronal maturation changes with age. Newborn DG neurons undergo faster neuronal maturation during the early postnatal period than in adulthood (Zhao et al., 2006), and aging further slows the lineage progression and neuronal maturation (Rao et al., 2005; Ngwenya et al., 2015; Trinchero et al., 2017, 2019). New dentate granule neurons play a unique role in the hippocampal circuitry as they traverse through an immature state in which they have physiologically distinct properties from mature neurons (Ge et al., 2007, 2008). As the maturation speed slows, newborn neurons remain in an immature state for increasingly longer periods of time, potentially expanding the amount of time that they uniquely impact the hippocampal circuit. Interestingly, experience and the cellular environment can acutely regulate the speed of neuronal maturation in adulthood. For example, neuronal maturation is accelerated by experiences, such as voluntary exercise and anti-depressant treatment with fluoxetine or electroconvulsive stimulation, or altered niche environment, such as inhibiting BMP or glucocorticoid signaling (Zhao et al., 2006; Wang et al., 2008; Ma et al., 2009; Fitzsimons et al., 2013; Jang et al., 2013; Bond et al., 2014; Trinchero et al., 2017). In addition, some regulators, such as Disrupted-in-Schizophrenia 1 (DISC1), regulate the speed of neuronal maturation at some ages but not others, suggesting that the tempo of neuronal maturation may be differentially regulated at different ages (Duan et al., 2007; Kim et al., 2012).

Though we have discussed how some of the known cellular properties that change across the lifespan impact levels of hippocampal neurogenesis, there are likely many more still undiscovered. Some age-dependent changes occur abruptly, such as a developmental event, while others gradually change over time more akin to the aging process. It is possible that some of the age-dependent changes act in a compensatory manner to extend the impact of each NSC and newborn neuron as the rate of neurogenesis declines across the lifespan.

## Progression of Hippocampal Neurogenesis Is Conserved Between Species, but Its Timing Is Species-Dependent

Adult hippocampal neurogenesis was originally discovered in rats (Altman and Das, 1965), and subsequent research suggests that protracted hippocampal neurogenesis occurs in the vast majority of mammals, including humans (Eriksson et al., 1998; Knoth et al., 2010; Spalding et al., 2013; Patzke et al., 2015; Hevner, 2016; Boldrini et al., 2018; Moreno-Jiménez et al., 2019; Tobin et al., 2019). While, the developmental progression of neurogenesis appears to be evolutionarily conserved, the timing of milestones is species-specific. Most neurogenesis research has been conducted in rat and mouse, which has biased the field to a rodent-centric view of hippocampal neurogenesis. Accurately comparing developmentally-equivalent ages between species will be critical to translate the large body of rodent model research to other mammalian species.

When hippocampal neurogenesis is viewed in the context of the entire lifespan (from conception to death), a similar progression emerges across species (Figure 1B; Amrein et al., 2011; Charvet and Finlay, 2018; Snyder, 2019). A rapid burst of neurogenesis occurs very early in the lifespan, generating most DG neurons, followed by a prolonged period of relatively low levels of neurogenesis that lasts for the rest of the lifespan. This pattern of neurogenesis is preserved across species, but the timing of the progression differs (Figure 1D). For example, hippocampal neurogenesis begins during the second half of gestation in rodent models (E10 in mouse and E14-20 in rat) and peaks around birth (Angevine, 1965; Schlessinger et al., 1975; Bayer, 1980a,b; Bond et al., 2020). As a result, most DG neurons are born postnatally. In contrast, peak neurogenesis occurs prenatally in species with longer gestation periods, such as spiny mice, guinea pigs, and non-human primates, so that most DG neurons are instead generated prenatally (Rakic and Nowakowski, 1981; Brunjes, 1984; Guidi et al., 2005). This shift in timing is further accentuated in humans where hippocampal neurogenesis begins in early gestation and peaks between the first and second trimester (Yang et al., 2014; Cipriani et al., 2017; Sorrells et al., 2018). As a result, the protracted period of relatively low-level neurogenesis begins much earlier in the lifespan of species with longer gestation times (Angevine, 1965; Schlessinger et al., 1975; Bayer, 1980a,b; Rakic and Nowakowski, 1981; Jabès et al., 2010; Sorrells et al., 2018).

Species-specific differences in hippocampal neurogenesis make it challenging to compare among species, especially later in the lifespan. Absolute numbers of newborn neurons are not comparable among species due to differences in the size of the brain and hippocampus (Patzke et al., 2015), but some studies have addressed this issue by measuring newborn DG neuron levels as a proportion of the total dentate granule cell number or as a percentage of maximum lifetime neurogenesis (Amrein et al., 2011; Charvet and Finlay, 2018; Snyder, 2019). Aligning developmentally-equivalent ages across species is complicated by differences in the timing of hippocampal neurogenesis. Using lifespan milestones, such as birth or puberty, to align hippocampal neurogenesis can be very misleading due to species-specific shifts in the timing of peak neurogenesis (Amrein et al., 2011; Charvet and Finlay, 2018; Snyder, 2019) and differences in the speed of neuronal maturation (van Praag et al., 2002; Laplagne et al., 2006; Ngwenya et al., 2006, 2015; Snyder et al., 2009; Kohler et al., 2011). Ideally, future comparative studies will align developmental milestones of the neurogenesis trajectory, such as peak neurogenesis. Correct alignment of the beginning of protracted low-level neurogenesis will be critical for accurate inter-species comparisons because neurogenesis continues to decline in a pattern of exponential decay during this period in multiple species, including humans (Figure 1D; Kuhn et al., 1996; Ben Abdallah et al., 2010; Knoth et al., 2010; Amrein et al., 2011; Moreno-Jiménez et al., 2019). These alignment strategies will require thorough time course analysis of DG neurogenesis across the lifespan of many different species, which is currently limited and fragmented, even in rodent models.

## Future Challenges and Perspectives in the Field of Hippocampal Neurogenesis

Hippocampal neurogenesis in the DG is a continuous, lifelong process that follows a similar developmental progression across mammalian species. The discovery that hippocampal neurogenesis persists into adulthood in rats (Altman and Das, 1965) has fueled decades of (mostly rodent) research, with the goal of promoting the endogenous capacity to generate new neurons for plasticity and regeneration throughout life. The natural, but naïve, assumption that human hippocampal neurogenesis exactly mirrors rodent studies has been challenged by recent studies suggesting that human neurogenesis is sparse and potentially drops to undetectable levels in adulthood (Sorrells et al., 2018; Franjic et al., 2022). The recent controversy over human hippocampal neurogenesis has highlighted long-held assumptions and gaps in our knowledge regarding neurogenesis across the lifespan and between species (Kempermann et al., 2018; Snyder, 2019; Seki, 2020; Moreno-Jimenez et al., 2021; Sorrells et al., 2021). Here we discuss future research directions that will fill these gaps and give us a more holistic perspective of hippocampal neurogenesis.

Hippocampal neurogenesis will be best studied as a life-long process due to its continuous nature. However, most research so far has been focused on a narrow slice of the lifespan. Rodent studies of hippocampal neurogenesis have overwhelmingly been focused on only ∼5–15% of the lifespan, which straddles the shift from peak neurogenesis to protracted low-level neurogenesis (Snyder, 2019). In contrast, most human studies used sample ages that were from >∼25% of the lifespan, well into the exponential decline that occurs in the protracted period of low-level neurogenesis (Snyder, 2019). As a result, rodent and human studies are likely studying very different stages of neurogenesis over the lifespan, which has contributed to discordant conclusions. Aging studies (Kuhn et al., 1996; Encinas et al., 2011; Harris et al., 2021; Ibrayeva et al., 2021) and studies exploring the link between developmental and adult neurogenesis (Li et al., 2013; Hochgerner et al., 2018; Berg et al., 2019; Noguchi et al., 2019; Borrett et al., 2022) have begun to compare neurogenesis across the lifespan. Going forward, we need more time course analyses across the lifespan to uncover age-dependent properties of hippocampal neurogenesis. Ideally, the development of non-invasive methods to repeatedly measure neurogenesis in the same subject across different life stages could reduce animal use and could potentially be applied to human subjects (Manganas et al., 2007; Ho et al., 2013; Tamura et al., 2016). Time course analyses in different species will identify conserved milestones in the neurogenesis progression and their species-dependent timing.

Once we have a thorough understanding of the developmental progression of hippocampal neurogenesis, we need to unpack the underlying mechanisms that orchestrate the sequence and tempo of developmental events (Ebisuya and Briscoe, 2018). For example, what initiates the NSC transition from proliferation to quiescence? Is there an intrinsic NSC program driven by a network of transcription factors or changes in the epigenome, or is there an extrinsic NSC program driven by signals from the developing environment? Illuminating the mechanisms regulating the NSC transition to quiescence will provide insight into how the capacity for long-term neurogenesis is maintained in the DG. Another unanswered question is what controls the tempo of DG neuronal maturation across the lifespan? Some studies have identified factors, such as experience and niche signaling pathways, that can transiently change the tempo of neuronal maturation in adulthood (Filippov et al., 2003; Zhao et al., 2006; Wang et al., 2008; Fitzsimons et al., 2013; Bond et al., 2014; Trinchero et al., 2017). However, it remains unknown whether similar or different mechanisms regulate the speed of neuronal maturation at different stages of the lifespan. There are also differences in the tempo of neuronal maturation across species (van Praag et al., 2002; Laplagne et al., 2006; Ngwenya et al., 2006, 2015; Snyder et al., 2009; Kohler et al., 2011), which could impact the progression and function of neurogenesis. The rate of protein decay and cell cycle length account for species differences in the tempo of motor neuron differentiation (Rayon et al., 2020) and a similar mechanism could be responsible for species-related differences in DG neuron maturation. To harness the endogenous capacity for neurogenesis we must first identify the mechanisms that regulate the natural progression of neurogenesis across the lifespan.

Studying hippocampal neurogenesis across species may help us identify both conserved and species-specific functional roles of persistent hippocampal neurogenesis. Although there is evidence that most mammals retain neurogenic capabilities, adult Cetaceans (dolphins, porpoises, and whales) apparently lack DG neurogenesis and exhibit unusual hippocampal features, including a small size relative to overall brain size and a loose architecture (Patzke et al., 2015; Hevner, 2016). In addition, persistent neurogenesis in adult Chiroptera (bats) appears to vary by species (Amrein et al., 2007; Gatome et al., 2010). These exceptions suggest that some mammals may have evolved adaptations that no longer require persistent hippocampal neurogenesis. The hippocampal circuitry is wired slightly differently across species (Bergmann et al., 2016), which could impact the influence of persistent neurogenesis on cognitive function. Research that investigates the function of persistent neurogenesis across species may reveal evolutionary reasons for species-specific differences in the timing and levels of neurogenesis. Analysis of persistent hippocampal neurogenesis in many non-model species is often limited to simple analyses using DCX and/or proliferation markers in adult animals of unknown-age (Patzke et al., 2015; Hevner, 2016). Future comparative studies should expand to include more thorough analyses across the lifespan in different species.

One goal of hippocampal neurogenesis research is to learn how to improve human health by promoting the endogenous capacity of the DG to generate new neurons across the lifespan. The current challenge is translating the bulk of neurogenesis research performed in rodents to humans, which is complicated by species-specific differences in the timing of neurogenesis. For example, peak neurogenesis in humans is shifted to very early in the lifespan, which means that the protracted period of low-level neurogenesis probably begins *in utero*, rather than in young adulthood as it does in rodents. Going forward, the hippocampal neurogenesis field should have an open mind when considering how previous rodent research might map onto a human timeline. It is possible that the bulk of neurogenesis research from young adult rodents is more applicable to human childhood, and that aging rodent studies might apply to human adulthood. There is a field-wide consensus that human hippocampal neurogenesis is sparse by middle age, but that does not mean it should be discounted. Even low levels of neurogenesis can be enhanced to improve hippocampus-dependent cognition in very old age through experience, such as exercise, and signaling modifications, such as inhibiting BMP signaling or infusion of young blood-borne factors (Kronenberg et al., 2006; Villeda et al., 2011, 2014; Meyers et al., 2016; Blackmore et al., 2021). This could be important in the context of human neurodegenerative diseases, which are associated with altered adult hippocampal neurogenesis (Moreno-Jiménez et al., 2019; Terreros-Roncal et al., 2021). Considering interspecies differences in neurogenesis within the context of the lifespan will be critical to designing future studies that will be relevant to promoting the neurogenic capacity of the human DG.

## Summary

Hippocampal neurogenesis is a continuous, lifelong developmental process. Historically, however, neurogenesis research has been separated into embryonic and adult neurogenesis. We propose a working model in which the continuous and lifelong nature of neurogenesis is shared across species, but the timing of common developmental milestones is shifted in a species-dependent manner. Future research that allows for accurate comparison of developmental stages across species and reveals mechanisms that regulate the timing of neurogenic processes across the lifespan will be critical to confirming this model and may motivate the field to reframe the meaning of “adult neurogenesis.”

## Data Availability Statement

The original contributions presented in the study are included in the article/supplementary material, further inquiries can be directed to the corresponding author/s.

## Author Contributions

AMB contributed to original text and figure. All authors contributed to final manuscript and approved the submitted version.

## Conflict of Interest

The authors declare that the research was conducted in the absence of any commercial or financial relationships that could be construed as a potential conflict of interest.

## Publisher’s Note

All claims expressed in this article are solely those of the authors and do not necessarily represent those of their affiliated organizations, or those of the publisher, the editors and the reviewers. Any product that may be evaluated in this article, or claim that may be made by its manufacturer, is not guaranteed or endorsed by the publisher.
